# The Presence of Testis Determines Aristolochic Acid-Induced Nephrotoxicity in Mice

**DOI:** 10.3390/toxins15020118

**Published:** 2023-02-01

**Authors:** Wei-Long Li, Babu J. Padanilam, Jinu Kim

**Affiliations:** 1 Interdisciplinary Graduate Program in Advanced Convergence Technology & Science, Jeju National University, Jeju 63243, Republic of Korea; 2 Department of Urology, Tisch Cancer Institute, Icahn School of Medicine at Mount Sinai, New York, NY 10029, USA; 3 Department of Anatomy, Jeju National University College of Medicine, Jeju 63243, Republic of Korea

**Keywords:** aristolochic acid, nephrotoxicity, acute kidney injury, orchiectomy, ovariectomy, sex difference

## Abstract

Aristolochic acid (AA) is notorious for inducing nephrotoxicity, but the influence of sex on AA-induced kidney injury was not clear. This study sought to investigate sex differences in kidney dysfunction and tubular injury induced by AA. Male and female mice were bilaterally orchiectomized and ovariectomized, respectively. Fourteen days after gonadectomy, the mice were intraperitoneally injected with AA (10 mg/kg body weight/day) daily for 2 days and sacrificed 7 days after the first injection. Body weight, kidney function, and tubular structure were assessed. When compared between male and female non-gonadectomized mice, AA-induced body weight loss was greater in male mice than in female mice. Functional and structural damages in male kidneys were markedly induced by AA injection, but kidneys in AA-injected female mice showed no or mild damages. Ovariectomy had no effect on AA-induced nephrotoxic acute kidney injury in female mice. However, orchiectomy significantly reduced body weight loss, kidney dysfunction, and tubular injury in AA-induced nephrotoxicity in male mice. This study has demonstrated that testis causes AA-induced nephrotoxic acute kidney injury.

## 1. Introduction

Sociocultural gender is an ideal construct that is forcibly materialized through time [1]; however, biological sex is a fundamental factor that is definitely related to the incidence and progression of various diseases [2], including hypertension [3], chronic kidney disease (CKD) [4], and acute kidney injury (AKI) [5]. Those sex differences in non-reproductive organs were generally attributed to gonadal hormones or sex chromosomes [6]. Especially, female sex is protective against ischemic AKI in rodents and hospitalized patients requiring renal replacement therapy [7]. Although it is also known that chemotherapeutic agents such as doxorubicine and cisplatin induce less nephrotoxicity in female rodents compared with male rodents, the influence of sex on nephrotoxic AKI is still unclear.

Aristolochic acid (AA) is a potent nephrotoxin derived from plants of the *Aristolochiaceae* family but has been used for medicinal purposes for over 2500 years in many countries today. AA is also an etiological agent in the clinical syndromes known as Chinese herb nephropathy (CHN) and Balkan endemic nephropathy (BEN) associated with CKD and urothelial malignancy [8,9]. The sex ratio in BEN is approximately 1:1, in contrast to the female preponderance in CHN because of the setting of a weight loss herbal remedy [10]. The rate of AKI in patients with AA nephropathy is high and is associated with kidney dysfunction, tubular atrophy, interstitial inflammatory cell infiltration, and progression to end-stage renal disease [11,12,13]. Currently, there are only a few studies on sex differences in kidney function and tubular structure in response to AA-induced nephrotoxic AKI. In this study, we sought to determine the effect of sex on AA-induced nephropathy.

## 2. Results

### 2.1. Female Mice Are More Resistant to AA-Induced Nephrotoxicity

**Figure 1:**
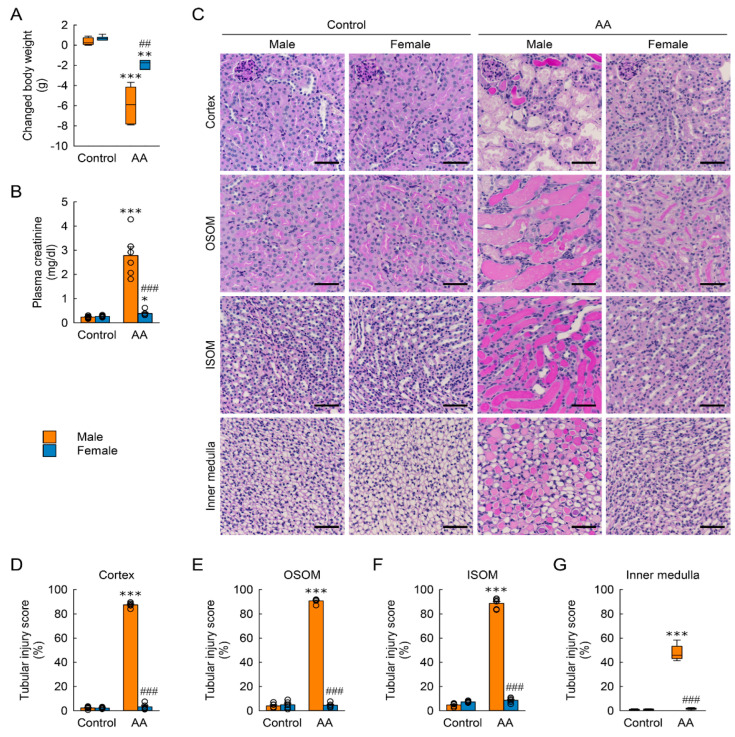
Sex differences in body weight change, kidney dysfunction, and tubular injury induced by aristolochic acid (AA). Mice were intraperitoneally injected with AA (10 mg/kg body weight/day) in 0.9% saline (control) for 2 days and sacrificed at 7 days after the first injection (*n* = 6 mice per group). (**A**) Body weight changes from 0 to 7 days after the first injection. H = 20.041, N_1–4_ = 6, *p* < 0.001. (**B**) Plasma creatinine concentration. The effect of AA: F_1,20_ = 192.511, *p* < 0.001; sex: F_1,20_ = 83.618, *p* < 0.001; interaction between AA and sex: F_1,20_ = 107.893, *p* < 0.001. (**C**) Representative images of periodic acid-Schiff (PAS)-stained kidneys including the cortex, outer stripe of the outer medulla (OSOM), inner stripe of the outer medulla (ISOM), and the inner medulla. In the cortex, the effect of AA: F_1,20_ = 4144.218, *p* < 0.001; sex: F_1,20_ = 3971.979, *p* < 0.001; interaction between AA and sex: F_1,20_ = 3963.988, *p* < 0.001. In the OSOM, the effect of AA: F_1,20_ = 2415.482, *p* < 0.001; sex: F_1,20_ = 2376.425, *p* < 0.001; interaction between AA and sex: F_1,20_ = 2464.488, *p* < 0.001. In the ISOM, the effect of AA: F_1,20_ = 1867.471, *p* < 0.001; sex: F_1,20_ = 1542.476, *p* < 0.001; interaction between AA and sex: F_1,20_ = 1749.155, *p* < 0.001. In the inner medulla, H = 15.867, N_1–4_ = 6, *p* = 0.001. Scale bar, 50 μm. (**D**–**G**) Tubular injury scores in the kidney cortex, OSOM, ISOM, and the inner medulla. Data are presented as the median value with quartiles (**A**,**G**) and the mean ± standard error of the mean with individual data points (**B**,**D**–**F**). A Kruskal-Wallis followed by Student-Newman-Keuls post hoc test (**A**,**G**) and 2-way analysis of variance followed by Tukey’s post hoc test (**B**,**D**–**F**) were used to determine statistical significance. * *p* < 0.05, ** *p* < 0.01, *** *p* < 0.001 versus control; ## *p* < 0.01, ### *p* < 0.001 versus male.

The sex differences in AA-induced nephrotoxicity were investigated by an evaluation of body weight loss in adult male and female mice at 6 days after two daily injections of AA or control. The AA-injected mice showed a significant loss of body weight compared with control mice (Figure 1A). When compared between male and female mice, the AA-induced body weight loss was markedly greater in male mice than in female mice (Figure 1A). To evaluate kidney dysfunction, plasma concentrations of creatinine were measured as a measure of glomerular filtration rate. The plasma creatinine concentration in control mice was below 0.3 mg/dL (Figure 1B). After an AA injection, plasma creatinine concentration increased significantly in male mice, but the increase in plasma creatinine concentration was modest in female mice (Figure 1B). To assess the structural damage to the kidneys, the kidney was divided into the cortex, OSOM, ISOM, and inner medulla and quantified by the following tubular injury parameters: composite of cast formation, tubular dilation, and sloughing of cells. Consistent with greater kidney dysfunction, male mice had higher scores of tubular injuries in the cortex, OSOM, ISOM, and inner medulla than did female mice after AA injection (Figure 1C–G). Among kidney regions in male mice, the tubular injury score in the inner medulla (47.8 ± 2.6%) showed less susceptibility to AA when compared with other scores in the cortex (88.7 ± 1.7%), OSOM (90.8 ± 0.8%), and ISOM (87.4 ± 0.8%) (Figure 1D–G). Intriguingly, female mice had no significant injury to tubules in all regions of the kidneys after AA injection when compared with that in control female mice (Figure 1C–G). In control kidneys, no significant difference was observed in body weight change, kidney function, or tubular structure between male and female mice.

### 2.2. Ovariectomy Has No Effect on AA-Induced Nephrotoxicity in Female Mice

**Figure 2:**
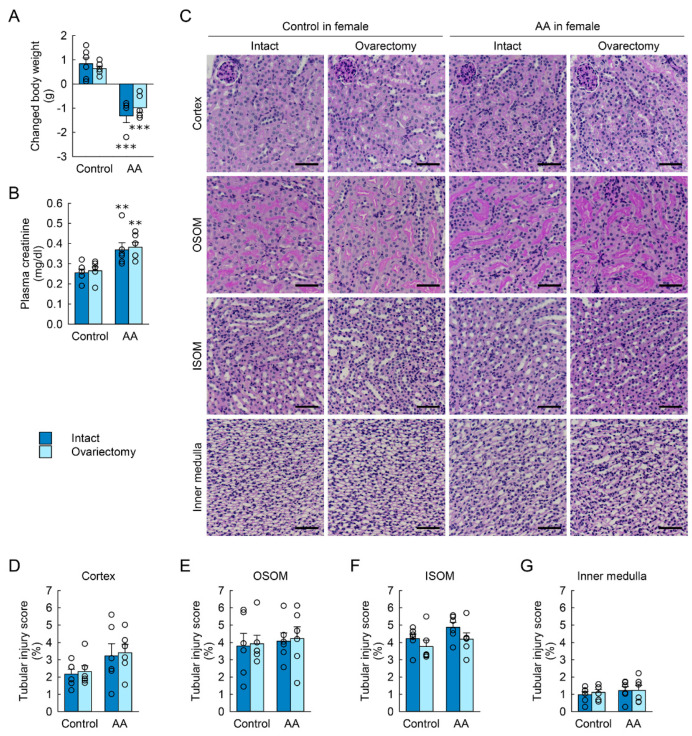
Effect of ovariectomy on body weight change, kidney dysfunction, and tubular injury induced by aristolochic acid (AA). Female mice were bilaterally ovariectomized or sham-operated (intact) at 14 days before the first injection, intraperitoneally injected with AA (10 mg/kg body weight/day) in 0.9% saline (control) for 2 days, and sacrificed at 7 days after the first injection (*n* = 6 mice per group). (**A**) Body weight changes from 0 to 7 days after first injection. The effect of AA: F_1,20_ = 76.233, *p* < 0.001; ovary: F_1,20_ = 0.0955, *p* = 0.760; interaction between AA and ovary: F_1,20_ = 1.528, *p* = 0.231. (**B**) Plasma creatinine concentration. The effect of AA: F_1,20_ = 20.486, *p* < 0.001; ovary: F_1,20_ = 0.211, *p* = 0.651; interaction between AA and ovary: F_1,20_ = 0.00430, *p* = 0.948. (**C**) Representative images of periodic acid-Schiff (PAS)-stained kidneys including the cortex, outer stripe of the outer medulla (OSOM), inner stripe of the outer medulla (ISOM), and the inner medulla. In the cortex, the effect of AA: F_1,20_ = 5.024, *p* = 0.036; ovary: F_1,20_ = 0.113, *p* = 0.740; interaction between AA and ovary: F_1,20_ = 0.000713, *p* = 0.979. In the OSOM, the effect of AA: F_1,20_ = 0.235, *p* = 0.633; ovary: F_1,20_ = 0.0564, *p* = 0.815; interaction between AA and ovary: F_1,20_ = 0.000451, *p* = 0.983. In the ISOM, the effect of AA: F_1,20_ = 2.708, *p* = 0.115; ovary: F_1,20_ = 2.985, *p* = 0.099; interaction between AA and ovary: F_1,20_ = 0.103, *p* = 0.751. In the inner medulla, F_1,20_ = 0.688, *p* = 0.417; ovary: F_1,20_ = 0.117, *p* = 0.736; interaction between AA and ovary: F_1,20_ = 0.0746, *p* = 0.788. Scale bar, 50 μm. (**D**–**G**) Tubular injury scores in the kidney cortex, OSOM, ISOM, and the inner medulla at 6 days after two daily injections of AA or control. Data are presented as the mean ± standard error of the mean with individual data points (**A**,**B**,**F**,**G**) and the median value with quartiles (**D**,**E**). A 2-way analysis of variance followed by Tukey’s post hoc test (**A**,**B**,**F**,**G**) and Kruskal-Wallis followed by Student-Newman-Keuls post hoc test (**D**,**E**) were used to determine statistical significance. ** *p* < 0.01, *** *p* < 0.001 versus control.

To determine whether ovaries affect the protection of kidney function and tubular structure from AA nephrotoxicity, bilateral ovariectomy was performed in female mice, and then body weight loss, kidney dysfunction, and structural damage induced by AA injection were investigated. Although the female mice showed significant loss of body weight and induction of plasma creatinine concentration after AA injection, no significant difference was observed in these parameters between ovariectomized and intact mouse kidneys (Figure 2A,B). Tubular injury scores in the cortex, OSOM, ISOM, and inner medulla were not significantly altered by AA and/or ovariectomy (Figure 2C–G).

### 2.3. Orchiectomy Reduces Susceptibility to AA-Induced Nephrotoxicity in Male Mice

**Figure 3:**
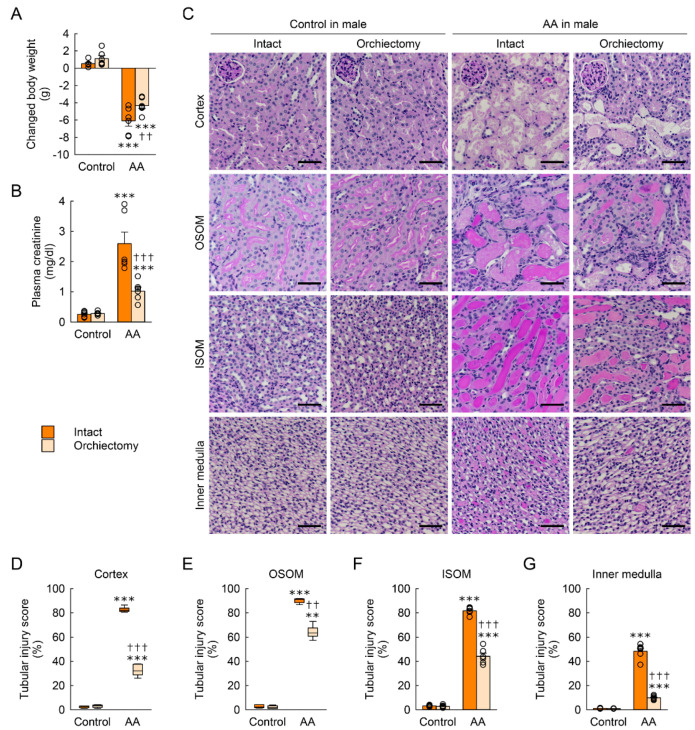
Effect of orchiectomy on body weight change, kidney dysfunction, and tubular injury induced by aristolochic acid (AA). Male mice were bilaterally orchiectomized or sham-operated (intact) at 14 days before the first injection, intraperitoneally injected with AA (10 mg/kg body weight/day) in 0.9% saline (control) for 2 days, and sacrificed at 7 days after the first injection (*n* = 6 mice per group). (**A**) Body weight changes from 0 to 7 days after the first injection. F_1,20_ = 220.808, *p* < 0.001; testis: F_1,20_ = 8.541, *p* = 0.008; interaction between AA and testis: F_1,20_ = 2.076, *p* = 0.165. (**B**) Plasma creatinine concentration. F_1,20_ = 152.069, *p* < 0.001; testis: F_1,20_ = 8.892, *p* = 0.007; interaction between AA and testis: F_1,20_ = 13.77300, *p* = 0.001. (**C**) Representative images of periodic acid-Schiff (PAS)-stained kidneys including the cortex, outer stripe of the outer medulla (OSOM), inner stripe of the outer medulla (ISOM), and the inner medulla. In the cortex, H = 19.767, N_1–4_ = 6, *p* ≤ 0.001. In the OSOM, H = 19.767, N_1–4_ = 6, *p* ≤ 0.001. In the ISOM, F_1,20_ = 1140.767, *p* < 0.001; testis: F_1,20_ = 19.027, *p* < 0.001; interaction between AA and testis: F_1,20_ = 6.756, *p* = 0.017. In the inner medulla, F_1,20_ = 1692.318, *p* < 0.001; testis: F_1,20_ = 137.022, *p* < 0.001; interaction between AA and testis: F_1,20_ = 94.764, *p* < 0.001. Scale bar, 50 μm. (**D**–**G**) Tubular injury scores in the kidney cortex, OSOM, ISOM, and the inner medulla at 7 days after two daily injections of AA or control. Data are presented as the mean ± standard error of the mean with individual data points. A 2-way analysis of variance followed by Tukey’s post hoc test were used to determine statistical significance. ** *p* < 0.01, *** *p* < 0.001 versus control; †† *p* < 0.01, ††† *p* < 0.001 versus intact.

To determine whether testes cause functional and structural damage after an AA injection, a bilateral orchiectomy was performed in male mice, and then body weight loss, kidney dysfunction, and structural damage induced by AA were assessed. Male mice showed a significant loss of body weight after an AA injection when compared with that in control mice (Figure 3A). When compared between orchiectomized and intact mice, the AA-induced body weight loss was significantly less in orchiectomized mice than in intact mice (Figure 3A). After an AA injection, plasma creatinine concentration increased markedly in intact mice, but the magnitude of the increase in creatinine level was significantly reduced in orchiectomized mice (Figure 3B). Consistent with increased kidney function, orchiectomized mice had significantly lower scores of tubular injuries in the cortex, OSOM, ISOM, and inner medulla than scores for intact mice after AA injection (Figure 3C–G). In control kidneys, no significant difference was observed in body weight change, kidney function, or tubular structure between orchiectomized and intact mice (Figure 3A–G).

## 3. Discussion

Many aspects of physiology and pathology differ between the sexes, but many animal studies still use a single sex, typically male. Furthermore, clinical studies often fail to include sex as a variable [2]. Thus, this failure to include sex as a biological variable has created a major knowledge gap. The current study provides important new information regarding sex differences in response to AA-induced nephrotoxicity. These results show, first, that AA injections into male mice markedly enhances body weight loss, kidney dysfunction, and tubular injury relative to control, but female sex abolishes them. Second, ovariectomy has no effect on the kidney function or tubular structure following exposure to AA. Third, orchiectomy is protective against functional and structural damages in AA-induced nephrotoxicity, as well as body weight loss.

The kidney is a highly complex organ formed by various types of tubular epithelial cells, often divided into segments based on morphology and localization. Because of that, under AKI conditions, those segments exhibit a broad spectrum of pathological responses [14]. Ischemic AKI predominantly affects the S3 segment of the proximal tubule located in the kidney OSOM [15,16], whereas septic AKI mainly does serious damage to the S1 to S3 segments of the proximal tubule in the kidney cortex and OSOM [17]. The results of most animal and clinical studies on AA-induced nephrotoxicity are limited to the cortex [12,18]. In the current study, AA-induced tubular damages spanned from the cortex through the outer medulla to the inner medulla in male kidneys, but they were not detected in female kidneys. Intriguingly, the inner medulla had less susceptibility to AA compared with other regions in the male kidney.

AA-linked pathological syndromes, especially BEN, are associated with local polymorphisms of cytochrome 450 [19]. AA also affects the cytochrome 450 metabolic pathway during the development of kidney injury and cancer [20]. Among the cytochrome P450 enzymes, CYP19A1, known as aromatase, converts androgen to estrogen [21] and can critically determine estrogen levels in males. Furthermore, previous studies have shown that estrogen steroid hormone is protective against AA-induced nephrotoxicity [22] and that its receptor is a target protein associated with AA [20]. Nonlyphenol as an environmental estrogen shows distinct toxicological effects in the sexes through sex-specific cytochrome P450 induction [23]. However, the detailed molecular mechanisms responsible for gonadal hormones and sex chromosomes in AA-induced nephrotoxicity remain to be explored.

It is well known that the risk for cardiovascular diseases in male and postmenopausal female patients is elevated [24], and the elevation is indispensable for the loss of estrogen [25]. Some kidney diseases also show an obvious difference between sexes in their incidence, progression, and response to treatment. Clinical and animal studies have shown that the male sex is much more susceptible to ischemic AKI when compared with the female sex [26,27]. Furthermore, orchiectomy reduces kidney susceptibility to ischemic AKI, whereas ovariectomy has no effect on the susceptibility [5]. Consistent with previous results in ischemic AKI, these results showed that AA-induced nephrotoxicity was seriously developed in male mice but not in female mice. Furthermore, when male and female mice were subjected to gonadectomy, the kidney injury was significantly reduced in male mice, but not altered in female mice. The overall mortality rate of AA-induced acute toxicity in male mice is higher than that in female mice [28]. Previous and current results suggest that the presence of testis, rather than the absence of the ovary, plays a critical role in the sex differences in kidney susceptibility to AA-induced nephrotoxicity. The results of this animal study should be compared with humans with caution. Previous studies have demonstrated that species-dependent differences in the toxicity between rodents and humans exist [29,30], which is a limitation of our study.

In conclusion, this study has shown that female mice have a lesser susceptibility to AA-induced nephrotoxicity, and that orchiectomy attenuates kidney functional and structural damage induced by AA. This finding provides a novel consideration of sexually dimorphic characteristics for protective strategies to avoid nephrotoxicity and AKI.

## 4. Materials and Methods

### 4.1. Animal Preparation

Eight- to ten-week-old C57BL/6 mice were purchased from Orient Bio (Seongnam, Gyeonggi, Republic of Korea). All animal experiments were performed in accordance with animal protocols approved by the Institutional Animal Care and Use Committee of Jeju National University under approval no. 2021-0045 on 13 July 2021. Mice maintained on a 12-h light/dark cycle 22 ± 2 ℃ with 55 ± 5% humidity and allowed free access to water and standard mouse diet chow. Mice were intraperitoneally injected with AA (10 mg/kg body weight/day; Sigma-Aldrich, St. Louis, MO, USA; product no. A9451) in 0.9% saline (control) daily for 2 days and sacrificed 7 days after the first injection. Male and female mice were bilaterally orchiectomized and ovariectomized, respectively [5], at 14 days before the first injection. Sham-operated mice underwent the same surgical procedure without gonadectomy. Before the surgeries and sacrifice, mice were anesthetized with an intraperitoneal injection of pentobarbital sodium (ENTOBAR; 60 mg/kg body weight; Hanlim Pharm, Seoul, Republic of Korea), as previously described [31].

### 4.2. Body Weight

Body weight was measured daily from 0 to 7 days after the first injection on an electronic digital scale accurate to 0.1 g (Ohaus, Parsippany, NJ, USA; product no. CS200).

### 4.3. Kidney Function

Under euthanasia with pentobarbital sodium (Hanlim Pharm), blood samples were obtained in heparinized syringes by cardiac puncture immediately before sacrifice. The samples were centrifuged at 9000 rpm for 10 min in a refrigerated Fresco 17 microcentrifuge (Thermo Fisher Scientific, Waltham, MA, USA; product no. 75002402). The supernatants were used as plasma to measure the creatinine concentration by an improved Jaffe method using the QuantiChrom creatinine assay kit (BioAssay Systems, Hayward, CA, USA; product no. DICT-500). The absorbance of plasma was measured at 510 nm using a SpectraMax i3x multi-mode microplate reader (Molecular Devices, San Jose, CA, USA, in the Bio-Health Materials Core-Facility, Jeju National University), as previously described [31].

### 4.4. Tubular Injury Score

Kidneys were fixed in 4% paraformaldehyde (Tech and Innovation, Chuncheon, Gangwon, Republic of Korea; product no. BPP-9016) for PAS stain. Tubular injury score was represented as a percentage of injured tubules that displayed cast formation, tubular dilation, and sloughing of cells in a blind manner under a Nikon Eclipse Ni microscope (Nikon, Tokyo, Japan) directly, as previously described [31]. The numbers of total and injured tubules were counted on 5 randomly chosen fields of ×400 magnification per kidney cortex: OSOM, ISOM, and inner medulla stained with PAS, respectively.

### 4.5. Statistical Analysis

All statistical analysis of the data was performed with SigmaPlot 14.0 software (Systat Software Inc., San Jose, CA, USA), as previously described [31]. The normal distribution was evaluated using a Shapiro-Wilk normality test. If the data did not show a normal distribution, a logarithmic transformation was attempted. Normally distributed data were analyzed with a 2-way analysis of variance (ANOVA) with a Tukey post hoc test. In F_α,β_ = γ for ANOVA, α, β, and γ represent degree of freedom for explained variance, degree of freedom for residual variance, and F value, respectively. Non-normally distributed data were analyzed with the Kruskal-Wallis test and a Student-Newman-Keuls post hoc test. In H = α, N_β_ = γ for Kruskal-Wallis test, α, β, and γ represent H value, group number, and sample size, respectively. In figures, normally and non-normally distributed data are presented as mean ± standard error of the mean (SEM) with individual data and median value with quartiles, respectively. A value of *p* < 0.05 were considered statistically significant.

## Data Availability

Raw data are available in Supplementary Materials.

## Supplementary Materials

The following supporting information can be downloaded at: https://www.mdpi.com/article/10.3390/toxins15020118/s1, Supplementary File S1: Raw data and statistical results.
